# Low-cord orchidectomy for testicular cancer: what would be different?

**DOI:** 10.1007/s00345-024-05118-7

**Published:** 2024-07-19

**Authors:** Ege A. Sarıkaya, Volkan Şen, Kutsal Yörükoğlu, Ozan Bozkurt

**Affiliations:** 1 Department of Urology, Dokuz Eylul University Faculty of Medicine, Izmir, Turkey; 2 Department of Pathology, Dokuz Eylul University Faculty of Medicine, Izmir, Turkey

**Keywords:** Testicular cancer, Low-cord orchidectomy, Sub-inguinal orchidectomy, Spermatic cord invasion

## Abstract

**Introduction:**

High cord radical orchidectomy (HRCO) is accepted as the standard surgical approach in testicular cancer, however low cord orchidectomy (LCRO) can reduce the morbidity of operation without worsening the oncological outcomes.

**Methods:**

We retrospectively re-examined the specimens of men to determine the level of spermatic cord invasion (SCI). Men who had proximal SCI with negative surgical margins after HRCO were assumed to have de-novo residual tumour if LCRO was performed. Others were assumed as oncologically similar. We examined the relation between pre-operative variables and SCI and proximal SCI to determine whether prediction of proximal SCI is possible.

**Results:**

196 patients were included. 22 (11%) had SCI and ten (5%) had proximal SCI. Four patients with proximal SCI had positive surgical margins even after HRCO and didn’t require additional local treatment. Six patients were assumed to have de-novo residual tumour if LCRO was performed. All six patients were metastatic and had systemic chemotherapy. High platelet count, tumour size, N stage, S stage and M stage were all significantly related with both SCI and proximal SCI (p < 0.05).

**Conclusion:**

Due to low probability of SCI, we think LCRO can safely be performed to reduce morbidity in Stage 1 patients. Although there is a risk for residual tumour in Stage 2–3 patients, currently there is no data that residual tumour would impair the success of systemic chemotherapy. Therefore we can not assume that these patients would be negatively affected. Pre-operative data can be useful to predict the presence of proximal SCI and select appropriate patients for LCRO.

## Introduction

Testicular cancer is the most common cancer in men aged between 20 and 35 with an incidence of 3.2/100000 [1]. It accounts for 1–2% of all male cancers and 95% are testicular germ cell tumours (TCGT) [2]. In TCGT, the initial step of both diagnosis and treatment is almost always radical orchidectomy. After orchidectomy, men will be referred to surveillance, chemotherapy, retroperitoneal lymph node dissection (RPNLD) or radiotherapy depending on the histopathological characteristics of the tumour, and presence of metastasis [3]. Due to advancements in current treatment options and guidelines, 5-year overall survival for TCGT is over 95% [4].

Preferred surgical approach is traditionally high-cord radical inguinal orchiectomy (HCRO), which involves excision of the testicle and spermatic cord up to the level of the internal inguinal ring [5]. In scrotal orchiectomy, the testicle and spermatic cord are excised at a more distal level. This approach is not recommended because it has worse lymphovascular control and oncological outcomes than HCRO [3]. In HRCO, an incision is made 2 cm superolateral to the pubic tubercle and the fascia of the external oblique muscle is incised to access to the inguinal canal. Then, the spermatic cord is reached within the inguinal canal and the fascia incision is extended between the external and internal inguinal ring (approximately 4–5 cm) to deliver the testicle [5]. After that, the spermatic cord is dissected up to the level of the internal ring and excised en-block with testis. Although HRCO is not related to mortality, it can result in long time morbidities and the need of subsequent operations.

The ilioinguinal nerve courses anterior to the spermatic cord within the inguinal canal and it carries the motor innervation of the transverse abdominis and internal oblique muscles, as well as the sensation of the scrotum, inner groin and roof of the penis [6]. If not well identified, ilıoinguinal nerve can be injured in inguinal canal during spermatic cord dissection. Injury of ilioinguinal nerve may lead to paresthesia, hyperesthesia, and persistent inguinal and scrotal neuralgia, which may not respond well to conservative treatment and may require surgery [4, 7, 8]. Phantom testis pain is reported to be as high as %25 after HRCO [9]. In addition, inguinal hernia may occur due to inappropriate repair of fascia or excessive dissection through inguinal canal floor [10]. Although it occurs in less than 1%, subsequent surgery is required for repair [5]. Nevertheless, HRCO traditionally remains as the standard technique for orchidectomy in TCGT.

**Fig. 1:**
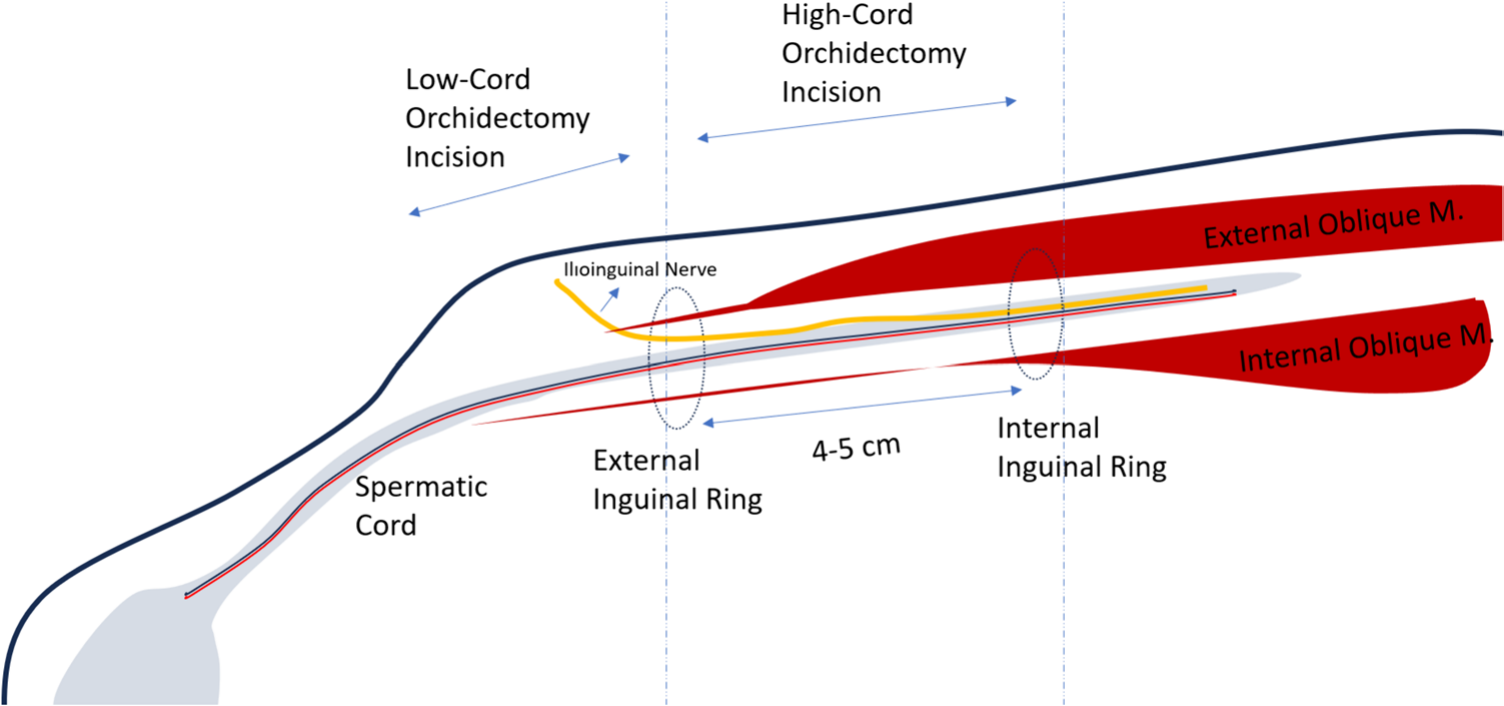
Incisions and anatomical landmarks of HRCO and LRCO

In low-cord radical orchidectomy (LCRO), spermatic cord is ligated at the level of the external inguinal ring instead of internal ring (Fig. 1). Some researchers have suggested that LCRO would eliminate the risk of inguinal hernia and ilioinguinal injury related morbidities without worsening oncologic outcomes [4, 8]. LRCO is less invasive than HCRO as subinguinal incision is sufficient, the external oblique fascia and inguinal canal is not disturbed and risk of ilioinguinal nerve injury and hernia are avoided. Since the length of the inguinal canal is approximately 4–5 cm, the length of the spermatic cord excised in LCRO is 4–5 cm shorter than HRCO [11]. Presence of tumour in the proximal 5 cm segment of spermatic cord would result in residual tumour and positive surgical margins after LCRO and these patients may not be appropriate for LCRO. However most patients with TCGT do not have proximal spermatic cord invasion (SCI) and LCRO would reduce morbidity and increase quality of life without compromising oncological outcomes for those patients.

We retrospectively examined the presence of tumour in the proximal spermatic cord of our HRCO specimens to identify the patients who would have residual tumour in case LCRO was carried out. And we evaluated the possible changes in additional treatment needs and oncological outcomes if LRCO was performed. We also investigated whether we can predict the presence of SCI and proximal SCI using pre-operatively available data.

## Methods

**Fig. 2:**
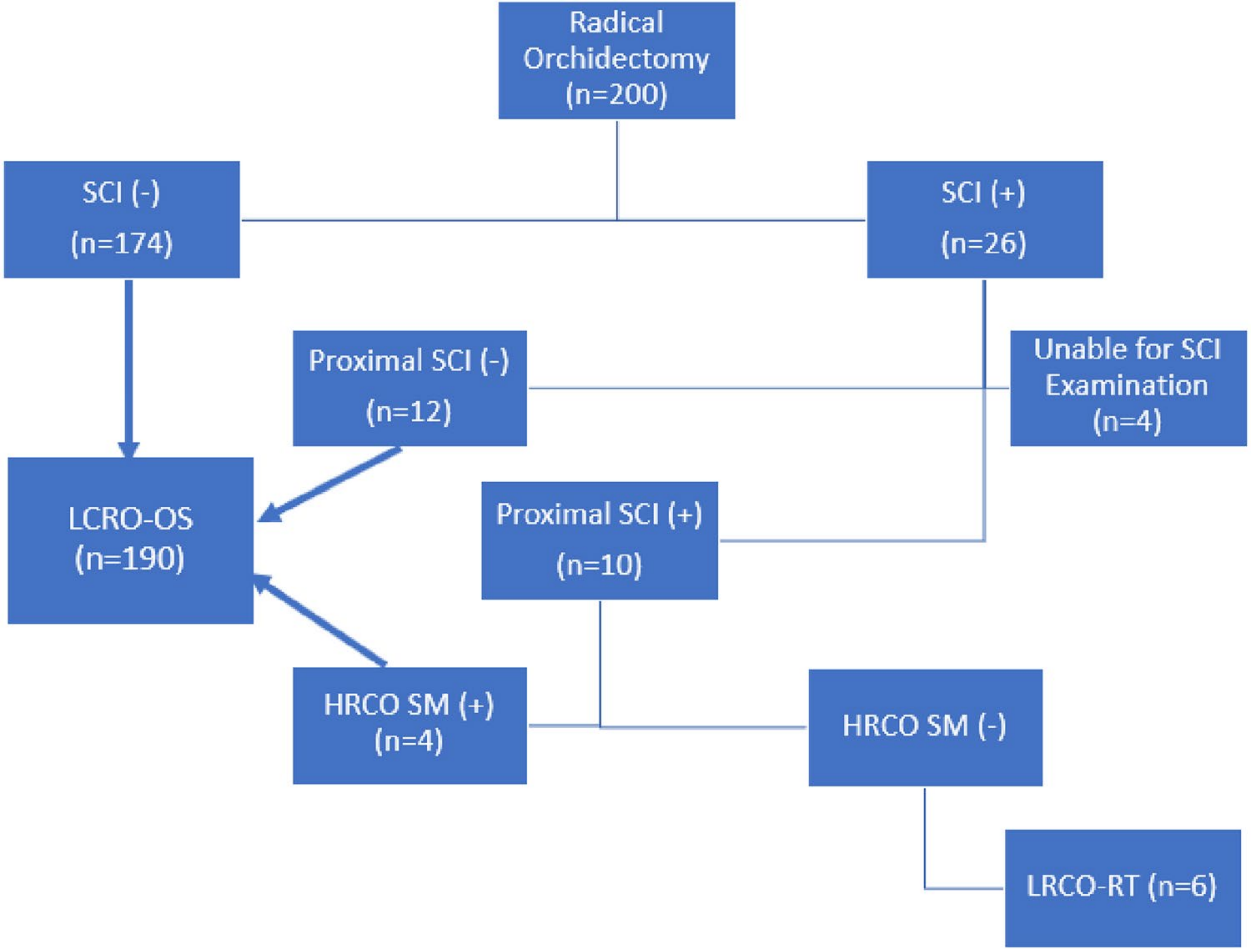
Grouping of patients according to spermatic cord and proximal spermatic cord invasions. *SCI* Spermatic cord invasion, *LCRO-OS* Similar outcome expected if LCRO was performed, *LCRO-RT* De-novo residual tumour if LCRO was performed, *HRCO SM* Surgical margins after HRCO

All patients with TCGT who underwent HCRO between 2010 and 2023 were included (n = 200). 26 of them had SCI. SCI (+) tissue specimens were re-examined to determine the level of SCI. Examination of 4 patients’ specimens could not define the exact level of spermatic cord invasion and these patients were excluded. Remaining 22 patients were examined for proximal SCI (5 cm) positivity. Patients who would have de-novo residual tumour in case of LCRO was performed were identified as patients who have tumour in proximal spermatic cord with negative HRCO surgical margins (LCRO-RT). Since patients with residual local tumour after HCRO (surgical margins (+)), would similarly have residual tumour after LCRO, these patients were assumed to be oncologically similar (LCRO-OS) along with patients with no proximal SCI (Fig. 2). We evaluated the treatments and oncological outcomes of LCRO-RT group and potential alterations if LCRO was performed.

We also compared pre-operatively available variables; (N stage, M stage, tumour size, tumour location, symptom, tumour markers, blood count parameters) between SCI positive and negative group and also between proximal SCI positive and negative group to investigate the probability of SCI and proximal SCI prediction pre-operatively. Nodal staging and presence of metastasis was determined by contrast-enhanced thoracoabdominal CT at the time of operation. Chi-square and Mann–Whitney U tests were used for univariate analysis and logistic regression was used for multivariate analysis in SPSS version 25.0. A p value < 0.05 was considered to be statistically significant.

## Results

Demographic, clinical and histopathological data are given in Table 1. The average follow-up duration of was 82.6 months (5–201 months). 174 (88.7%) SCI (–) men, 4 (2.0%) men with positive surgical margins after HRCO and 12 (6.2%) SCI (+) men with negative proximal SCI were assumed to be oncologically similar (Fig. 2). 6 (%3.1) proximal SCI (+) men with negative surgical margins after HRCO formed the LCRO-RT group.

**Table 1:** Clinical, histological and demographic data of patients with TCGT

	All Patients (196)	SCI (+) (22)	Proximal SCI (+) (10)
Age, mean (± std)	30.2 (± 8.2)	32.73 (± 9.7)	33.2 (± 10.2)
Symptom of Presentation, % (n)			
Mass	66.8 (131)	68.2 (15)	50 (5)
Pain	24.5 (48)	22.7 (5)	40 (4)
Infertility	3.1 (6)	9.1 (2)	10 (1)
Other	5.6 (11)	0 (0)	0 (0)
Duration of Symptoms, median (min–max)	30 (1–730)	145 (1–540)	60 (30–90)
Seminoma, % (n)	45.9 (90)	36.4 (8)	16.6 (1)
ns-TGCT, % (n)	54.1(106)	63.6 (14)	83.3 (5)
Mixed ns-TGCT	41.8 (82)	40.9 (9)	33.3 (2)
Embryonal Ca	7.7 (15)	18.2 (4)	50 (3)
Yolk Sac Tm	1 (2)	4.5 (1)	0 (0)
Teratoma	2.6 (5)	0 (0)	0 (0)
Choriocarcinoma	1 (2)	0 (0)	0 (0)
Tumour Size (mm)^a,b^	45 (2–160)	60.5 (25–160)	50 (30–130)
Side of Tumour, right % (n)	51.5 (101)	72.7 (16)	70 (7)
SCI ( +), % (n)	11.2 (22)	22 (100)	10 (100)
Surgical Margins ( +), % (n)	2 (4)	18.2 (4)	40(4)
Vascular Invasion ( +), % (n)	21.4 (42)	45.5 (10)	60 (6)
T Stage, % (n)			
1	65.3 (128)	0 (0)	0 (0)
2	23 (45)	0 (0)	0 (0)
3	11.7 (23)	100 (22)	100 (10)
4	0 (0)	0 (0)	0 (0)
N Stage, % (n)^a,b^			
0	51 (100)	9.1 (2)	10 (1)
1	23 (45)	40.9 (9)	10(1)
2	17.3 (34)	45.5 (10)	70 (7)
3	8.7 (17)	4.5 (1)	10 (1)
M Stage, % (n)^a,b^			
0	86.2 (169)	63.6 (14)	40 (4)
1a	11.2 (22)	22.7 (5)	40 (4)
1b	2.6 (5)	13.6 (3)	20 (2)
S Stage, % (n)^a,b^			
0	35.7 (70)	22.7(5)	0 (0)
1	44.4 (87)	40.9 (9)	60 (7)
2	15.8 (31)	27.3 (6)	20 (2)
3	4.1 (8)	9.1 (2)	10 (1)
UICC Stage, % (n)			
1	51 (100)	9.1 (2)	0 (0)
2 A/B	24.5 (48)	45.5 (10)	40 (4)
2 C	2.6 (5)	0 (0)	0
3	21.9 (43)	45.5 (10)	60 (6)
Adjacent Treatment, % (n)^a,b^			
Surveillance	31.1 (61)	0 (0)	0 (0)
Single-Dose CT	15.8 (31)	4.5 (1)	0 (0)
2 cycles BEP	6.1 (12)	9.1 (2)	0 (0)
3–4 cycles BEP	45.4 (89)	86.4 (19)	100 (10)
RT	1 (2)	0 (0)	0 (0)
RPNLD	4.6 (9)	0 (0)	0 (0)
Recurrence, % (n)	7.1 (14)	4.5 (1)	0 (0)
Mortality, % (n)	3.1 (6)	9.1 (2)	10 (1)

*TCGT* Testicular germ cell tumour, *ns-TCGT* non-seminomatous testicular germ cell tumour, *SCI* spermatic cord invasion

^a^Significantly different between SCI ( +) and SCI (–) groups

^b^Significantly different between proximal SCI ( +) and SCI (–) groups

Of the 6 patients, 1 had seminoma and 5 had non-seminomatous TCGT (ns-TCGT). The average tumour size was 49.8 mm (30–90 mm). 1 patient was classified as stage 2b and 5 patients were classified as stage 3 according to UICC prognostic groups (UICC, 2016, 8th edition) [12]. All 6 patients were given chemotherapy (3 or 4 cycles of BEP) and no relapse or mortality was observed.

### Positive vs. negative spermatic cord invasion

22 patients formed the SCI (+) group, 174 patients formed the SCI (–) group. Age, symptom of presentation, symptom duration, tumour side, TCGT subtype were similar. The average follow-up period of SCI (+) patients was 98 months (8–194 months) and the average length of the excised spermatic cord was 8.2 cm (5–14 cm). 4 patients (18%) had discontinuous SCI and 18 (82%) had continuous SCI.

In univariate analysis, a relationship was found between tumour size and SCI positivity (p = 0.005; 68.7 mm vs 42.3 mm). There were 45 patients with a tumour diameter below 25 mm, and none of these patients had SCI. Platelet count (PLT) was also significantly higher in SCI (+) group (p = 0.04, 256k vs 305k) while no relationship was found between AFP, LDH, HCG, Neu/Lymp ratio, Lymp/PLT ratio, Neu/PLT ratio, MPV and SCI positivity. N stage, M stage, S stage and the need for subsequent treatment were also related with SCI positivity (p = 0.00 for all). In multivariate regression analyses of pre-operative variables (clinical, staging and laboratory values), only M stage and tumour size were associated with the presence of SCI (p = 0.01 and 0.00, respectively). No difference was observed in terms of relapse or survival between groups (p > 0.05).

### Positive vs. negative proximal spermatic cord invasion

Ten patients were proximal SCI (+) and 186 were proximal SCI (–). Age, presenting symptom, symptom duration, tumour side and follow-up duration were similar. NS-TCGT rate was higher in the proximal SCI (+) group (p = 0.02; 54% vs 83%) (Table 1). No patients in proximal SCI (+) group had local disease (N0M0) or negative tumour markers (S0). 4 patients were classified as Stage 2b and 6 patients were classified as Stage 3 according to UICC, and all patients were given 3 or 4 cycles of BEP (Table 1).

PLT and tumour size were higher in proximal SCI (+) group (p = 0.09; 258 k u/L vs 322 k u/L and p = 0.049; 44.4 mm vs 61.2 mm, respectively) in univariate analysis. No patients with a tumour diameter lower than 3 cm (n = 51) had proximal SCI and interestingly, proximal SCI positivity was not observed in any patient with PLT below 210.000/uL (n = 53). N stage, M stage, S stage and the need for subsequent treatment was associated with proximal SCI positivity (p = 0.000, p = 0.000, p = 0.032 and p = 0.05 respectively).

In multivariate analyses, there was a relationship between N stage, M stage, platelet count and proximal SCI (p = 0.16, p = 0.00, p = 0.29 respectively). No relation was found between histopathological, clinical and other laboratory parameters. In the model created by using N stage, M stage, tumour size and laboratory data, the positive predictive value for the presence of proximal SCI was 80% and the negative predictive value was 96.8% (sensitivity 40%, specificity 99.5%, R square: 0.542).

## Discussion

### Oncological outcomes

The traditional standard surgical approach for TCGT is HRCO. However, recent studies suggest that low-cord radical orchiectomy can minimize the morbidity of orchidectomy without affecting oncological outcomes. In a retrospective study, the relapse rates and survival of patients who underwent LCRO and HRCO were similar, although only Stage 1 patients (according to Royal-Marsden Staging) were included in analysis due to low number of stage 2–4 patients [8]. It was also stated that the relapse percentage was higher in the HCRO arm, but the time until relapse was shorter in the LCRO arm in Stage 2–4 patients. As a result, it was suggested that LCRO can be recommended safely in Stage 1 patients. In another study containing 121 patients, rates of SCI and proximal SCI in TCGT were found as low as 4% and 0% respectively. Based on low rate of SCI, authors suggested that LCRO would be a less invasive alternative to HRCO with comparable oncological outcomes [7]. Other studies demonstrated similarly low SCI rates between %4 and 8 in TCGT [13–15]. Although proximal SCI was not evaluated and subgroup analysis according to stage was not present in most of the studies; proximal SCI in Stage 1 patients is expected to be even lower.

In our 13-year experience, proximal SCI was present in only 10 cases and none of these cases were classified as Stage 1 (T3-4N0M0S0). Likewise, proximal SCI was not observed in cases with a tumour diameter less than 3 cm. Since the probability of proximal SCI is very low in these two patient groups, we think that LCRO can be safely performed in stage 1 patients and in patients with small testicular masses.

Another controversial issue is whether spermatic cord surgical margin positivity affects survival in metastatic patients who will receive systemic chemotherapy. There is no clear evidence that local residual tumour lowers the success of systemic chemotherapy. Currently, international guidelines do not state any recommendation regarding the addition of further local treatment to systemic chemotherapy in the presence of positive surgical margins in metastatic TCGT [3, 16]. In our study 4 patients had positive spermatic cord margins after HRCO, all were metastatic and received chemotherapy. Three of them had complete response and one patient showed rapid progression and died. Although these patients had positive spermatic cord surgical margins, there were no local recurrence or need for additional local treatment, meaning systemic chemotherapy was also successful for local residual tumour control. In our study, an additional 6 patients would have de-novo residual tumour if LCRO was performed and all were metastatic and received systemic chemotherapy. No recurrence or mortality was observed in these 6 patients. However, it is not possible to say that these patients would have worse oncological outcomes due to residual local tumour, considering that all of them received systemic chemotherapy.

RPNLD can also be performed in N + patient group. Anderson et al. reported their experience of residual spermatic cord excision simultaneously with RPNLD in a patient with residual tumour in spermatic cord after LCRO. There were no surgical difficulty in residual spermatic cord removal [4].

Although discontinuous involvement of spermatic cord is accepted as metastatic disease according to The 8th Edition of the American Joint Committee on Cancer Staging Manual, 2 of our 4 patients were M1a and 2 were N2M0 in discontinuous involvement group and no relapse or mortality was observed.

### Morbidity

Complications such as inguinal hernia, hypoesthesia in the scrotum and groin, and persistent scrotal and inguinal neuralgia may occur after HRCO [7]. Chronic scrotal pain is a result of compression or damage of the relevant nerves. There is a risk of injury of ilioinguinal nerve in operations in which inguinal canal is opened, as ilioinguinal nerve runs close to the spermatic cord within the canal [4, 17]. Scrotal neuralgia in young males may not respond to conservative treatment, may require subsequent surgery, and may negatively affect the individual sexually or psychologically [18]. Incidence of chronic neuralgia after HRCO can be as high as 25% [5, 9]. On the other hand, no scrotal neuralgia were reported after 42 LCRO [4]. In another study including 222 patients with both HCRO and LCRO arms, there were no significant complications in LCRO arm and it was suggested that LCRO would reduce morbidity [8]. Operation time is also shorter in LCRO and it can be performed with a smaller incision [8].

### Predicting the spermatic cord and proximal spermatic cord invasion and patient selection

Various laboratory parameters (Hg, Hct, PLT, LYM, Neu, Neu/Platelet, Lymp/Platelet, mean platelet volume (MPV), AFP, LDH, B-HCG) have been evaluated as prognostic factors in TCGT and various results have been reported. Interestingly, we observed that high PLT was associated with both SCI and proximal SCI. Solid organ tumours can elevate PLT by increasing proinflammatory cytokines such as IL-6 and one previous study demonstrated that PLT > 400.000 u/L is related to residual disease and spermatic cord invasion and might serve as a prognostic factor in TCGT [19, 20]. Although low MPV was previously suggested as a poor prognostic indicator for TCGT, we didn’t find a relationship between MPV and survival or SCI [21]. While no relationship was present between tumour markers (AFP, B-HCG and LDH) and SCI; tumour diameter, platelet count, N stage and M stage was significantly related with both SCI and proximal SCI. With the model created by these variables’ multivariate analysis, we can help the prediction of the proximal SCI and enable LCRO to be recommended more safely.

## Conclusion

We think that LCRO is a safe and feasible approach in stage 1 patients since the likelihood of proximal SCI is low. In stage 2–3 patients, either systemic chemotherapy or RPNLD (± spermatic cord excision if positive) will be administered; thus we can’t say that the presence of residual tumour in the spermatic cord will definitely affect the oncological results. We showed that pre-operative variables can predict the presence of proximal SCI and may be guide to safely recommend LCRO to proximal SCI (–) patients to reduce comorbidity. In order for LCRO to be used as the standard method in a subgroup of TCGT patients with low proximal SCI prediction, long-term studies with comparable cancer-specific and overall survival outcomes are needed.

## Data Availability

The datasets generated during and/or analysed during the current study are available from the corresponding author on reasonable request.
